# Metabolic Engineering a Model Oilseed *Camelina sativa* for the Sustainable Production of High-Value Designed Oils

**DOI:** 10.3389/fpls.2020.00011

**Published:** 2020-02-12

**Authors:** Lixia Yuan, Runzhi Li

**Affiliations:** ^1^College of Biological Science and Technology, Jinzhong University, Jinzhong, China; ^2^Institute of Molecular Agriculture and Bioenergy, Shanxi Agricultural University, Taigu, China

**Keywords:** *Camelina sativa* (L.) Crantz, model oilseed, metabolic engineering, fatty acids, designed oil

## Abstract

*Camelina sativa* (L.) Crantz is an important Brassicaceae oil crop with a number of excellent agronomic traits including low water and fertilizer input, strong adaptation and resistance. Furthermore, its short life cycle and easy genetic transformation, combined with available data of genome and other “-omics” have enabled camelina as a model oil plant to study lipid metabolism regulation and genetic improvement. Particularly, camelina is capable of rapid metabolic engineering to synthesize and accumulate high levels of unusual fatty acids and modified oils in seeds, which are more stable and environmentally friendly. Such engineered camelina oils have been increasingly used as the super resource for edible oil, health-promoting food and medicine, biofuel oil and high-valued chemical production. In this review, we mainly highlight the latest advance in metabolic engineering towards the predictive manipulation of metabolism for commercial production of desirable bio-based products using camelina as an ideal platform. Moreover, we deeply analysis camelina seed metabolic engineering strategy and its promising achievements by describing the metabolic assembly of biosynthesis pathways for acetyl glycerides, hydroxylated fatty acids, medium-chain fatty acids, ω-3 long-chain polyunsaturated fatty acids, palmitoleic acid (ω-7) and other high-value oils. Future prospects are discussed, with a focus on the cutting-edge techniques in camelina such as genome editing application, fine directed manipulation of metabolism and future outlook for camelina industry development.

## Introduction

Plant seed oils enriched in triacylglycerols (TAGs) consisting of three fatty acids esterified to a glycerol backbone are energy-dense molecules that are utilized for energy production in the life cycle of plants (Athenstaedt and Daum, 2006). More importantly, they not only provide the nutritional requirements of humans and animals but also serve as a renewable chemical feedstock for biofuels and various industrial applications (van Erp et al., 2011). Although global production of vegetable oils has increased in recent decades, a wider gap between the production and consumption still exists. To meet the ever-growing market demands for vegetable oils, it is much needed to genetically improve seed oil yield and quality from oil crops.

The fatty acid profile and their distribution in TAGs of plant oils determines oil quality, physicochemical properties, and uses. TAGs from commercially grown oilseed crops typically contain mainly five fatty acids including palmitic (16:0), stearic (18:0), oleic (18:1Δ9), linoleic (18:1Δ9,12), and a-linolenic (18:1Δ9,12,15) acids. In contrast, a wide variety of fatty acids with different chain lengths and functional groups were found to be highly accumulated in seeds of many uncultivated plant species (Badami and Patil, 1980). TAG containing modified fatty acids with functionality beyond those found in commercially-grown oil seed crops represents a valued resource for bio-based materials and other diverse uses (Dyer et al., 2008).

Over the years, an increased understanding has been made on plant lipid metabolism and its regulation, coupled by well characterization of fatty acid biosynthesis, modification and assembly into TAGs (Cahoon et al., 2002; Kagale et al., 2014). With this information and the wealth of genetic diversity for synthesis of novel fatty acids and storage oils, plant seeds have been developing as platform for the design and tailoring of biochemical pathways to synthesize diverse nutritional and industrial oils not currently found in oilseed crops (Haslam et al., 2016). Various genetic modification tools have been developed including gene editing and synthetic biology techniques, which allow to rapidly assembly novel pathways in oilseed crops for commercially producing high levels of designed lipids/oils and high-valued compounds.

Until recently, much of this work was made in metabolically engineering the model plant *Arabidopsis thaliana* for the production of various modified fatty acids (Arondel et al., 1992; Farmer et al., 1998; Mu et al., 2008; Bates and Browse, 2011). However, this model plant has poor agronomic traits such as small seed yield and unable large-scale field cultivation, which has limited the functional testing of the modified oil. In contrast, *Camelina sativa* (L.) Crantz, an important oilseed crop in the family Brassicaceae, possesses a number of valuable agronomic traits that recommend it as both a new model system and an ideal crop platform for lipid metabolic engineering (Zubr, 1997; Kagale et al., 2014; Ruiz-Lopez et al., 2015; Bansal and Durrett, 2016; Malik et al., 2018). Camelina has a relatively short life cycle, low water and fertilizer requirements. Camelina seed yield is comparable to other oil seed crops, particularly under stress conditions. It's simple, effective transformation system, combined with the availability of abundant transcriptomic and genomic data, has allowed the generation of engineered camelina lines capable of synthesizing high levels of novel oils or UFAs, further enabling subsequent field testing of such traits at a large scale (Lu et al., 2011; Nguyen et al., 2013; Ruiz-Lopez et al., 2014; Malik et al., 2018).

This review was conducted to investigate why camelina is particularly attractive as an ideal model oilseed for metabolic engineering and a platform for commercial production of high-valued bioproducts. We will briefly overview advances in the metabolic engineering of unusual lipids or novel oils in this oil seed crop, combined with author group's work on camelina functional genomics and genetic improvement (Li et al., 2010; Wu et al., 2012; Yuan et al., 2017a; Yuan et al., 2017b). Main description in the selected examples focus on the pathway reconstruction for high synthesis and accumulation of acetyl triacylglycerols, hydroxylated fatty acids, medium-chain fatty acids, ω-3 long-chain polyunsaturated fatty acids, ω-7 monounsaturated fatty acids, and other novel lipids having beneficial functional groups or properties. Moreover, we discuss the cutting-edge research directions in camelina such as genome editing application, a flexible and useful substrate for applied synthetic biology, and future outlook for camelina industry development.

## An Ideal Model Oilseed for Lipid Metabolic Engineering

Camelina has been identified as a promising new crop for oil production due to its several excellent characteristics of low requirements, a short crop cycle (80–100 days), high disease-pest resistance and stress tolerance (Zubr, 1997; Zanetti et al., 2013). In terms of performance, camelina has high yield in favorable environments, and camelina seeds accumulate high levels of oil (40%) and protein (30%) compared to other Brassicaceae crops (Vollmann and Eynck, 2015). Particularly, in camelina oil, UFAs make up 90%, including 40% of a-linolenic acid (ω-3), 25% of linoleic acid, 15% of oleic acid, and 15% of eicosenoic acid. This desirable fatty acid composition enables camelina to be developed as nutritionally enhanced oils. With several agronomic advantages, camelina could be easily developed for commercial production of vegetable oil as much healthy food and a renewable resource for green manufacture of high-quality biofuels (Lu and Kang, 2008).

Meanwhile, camelina has been considered as a platform for the production of specific oils (Collins-Silva et al., 2011; Bansal and Durrett, 2016; Haslam et al., 2016). Camelina shares many (> 90%) of the genes involved in lipid metabolism with the genetic model plant Arabidopsis (Nguyen et al., 2013; Kagale et al., 2014). The rational design of lipid pathways in camelina had made more reliable with the reference genome in 2014 (Kagale et al., 2014). Moreover, the expanded lipid gene family in *C. sativa* provides greater diversity in enzyme expression and substrate specificity. Many of the shortcomings associated with model species, discussed later, can be overcome with camelina, as it has the ability to be both an experimental model system and recognized oilseed crop. Currently, camelina is getting the rising interest and increasing expanding of cultivation across the world (Gugel and Falk, 2006; Mcvay and Khan, 2011; Guy et al., 2014; Malik et al., 2018). A number of lipid metabolic engineering in seeds of *C. sativa* in recent years were summarized in **Table 1**.

**Table 1:** Lipid metabolic engineering in seeds of *Camelina sativa* in recent years.

Target lipid	Target gene	Manipulation	Phenotype	Reference
Cyclopropane fatty acids (CPAs)	Lychee phosphatidylcholine: diacylglycerol cholinephosphotransferase (LcPDCT) and Escherichia coli cyclopropane synthase (EcCPS)	Co-overexpression	50% increase of CPAs	Yu et al. (2019)
Medium-chain FA (MCFA)-containing acetyl-TAGs (MCFA-AcTAGs)	EaDAcT, ChFatB2, cpFatB2, UcFatB1, CnLPAAT, CsDGAT1, and CsPDAT1	Co-expression of EaDAcT with one or two of ChFatB2, cpFatB2, UcFatB1, CnLPAAT, plus combination with CsDGAT1-RNAi and CsPDAT1-RNAi	Significant increased levels of MCFA-AcTAGs, MCFA-AcTAGs was up to 77% more in the best lines	Bansal et al. (2018)
Acetyl-TAGs (AcTAGs)	EaDAcT, and CsDGAT1	Co-expression of EaDAcT together with RNAi suppression of CsDGAT1	AcTAGs with a 2-fold reduction in very long chain fatty acids was up to 85 mol % in the field-grown transgenic line	Liu et al. (2015a)
Saturated FAs, Oleic acid	Fatty acyl-ACP thioesterases (FATB)	Artificial microRNA mediated CsFATB gene suppression (amiFATB)	35% reduction of total saturated FAs and an increase of oleic acid in seed oil	Ozseyhan et al. (2018)
Hydroxy fatty acids (HFAs)	Lesquerella (*Physaria fendleri*) fatty acid elongase (LfKCS3) and castor fatty acid hydroxylase (RcFAH12)	Co-expression of LfKCS3 and RcFAH12	Increased HFAs higher than that in the transgenics expressing RcFAH12 alone	Snapp et al. (2014)
C(20-C24 very long chain FAs (VLCFAs,), C18 unsaturated FAs	Fatty acid elongase1 (FAE1)	Knocking out three CsFAE1 alleles by CRISPR technology with an egg cell-specific Cas9 expression	Reduction of VLCFAs from over 22% to less than 2% of total FAs and concomitant increase of C18 unsaturated FAs	Ozseyhan et al. (2018)
Hydroxy fatty acids (HFAs)	Phospholipase C-like protein (RcPLCL1) and fatty acid hydroxylase (RcFAH12) from *Ricinus communis*	Seed-specific coexpression of RcPLCL1 and RcFAH12	HFAs was increased to 24% of total FAs	Aryal and Lu (2018)
Oleic acid	Fatty acid desaturase 2 (FAD2)	Knockout of all three CsFAD2s by CRISPR/Cas9	Oleic acid level was increased from 16% to >50% and total MUSFA (18:1, (20:1, 22:1) increased from 32% to >70% with concurrent decreases in linoleic and linolenic acids	Jiang et al. (2017)
Oleic acid	Fatty acid desaturase2 (FAD2)	Knockout of three, two, and an isologous CsFAD2 by CRISPR/Cas9	Oleic acid level was increased from 10% to 62% of total FAs in different allelic combinations	Morineau et al. (2017)
Linolenic(18:3) and eicosenoic acids (20:1)	Two Arabidopsis phospholipase Dζ genes (AtPLDζ1 and AtPLDζ2 )	Co-expression of AtPLDζ1 and AtPLDζ2	TAG was increased by 2% to 3% more compared to wild type. Increase of 18:3 and (20:1 FAs was concurrent with decrease of other FAs	Yang et al. (2017)
Oil and seed yields	Arabidopsis diacylglycerol acyltransferase1 (AtDGAT1) and a yeast cytosolic glycerol-3-phosphate dehydrogenase (ScGPD1)	Seed-specific coexpression of AtDGAT and ScGPD1	The transgenic seeds showed up to 13% higher seed oil content and up to 52% increase in seed mass, with decreased 18:1 level	Chhikara et al. (2018)
Linolenic(18:3) and linoleic acid (18:2)	CsPDAT1 and CsDAT1	Overexpression of CsPDAT; silencing of CsDGAT1 by amiRNA	Levels of 18:3 was up to 56 mol% of total FAs in CsDGAT1-silenced lines; Levels of 18:2 was increased with concurrent decrease of 18:3 in the CsPDAT-overexpressed lines.	Marmon et al. (2017)
α-linolenic acid, linoleic acid	microRNA167A (miR167A), camelina fatty acid desaturase3 (CsFAD3) and, auxin response factor 8 (CsARF8)	Seed-specific expression of miR167 which suppresses CsARF8 and then mediates transcriptional cascade for CsFAD3 suppression via the ABI3-bZIP67 pathway	Decrease of α-linolenic acid and concomitant increase of linoleic acid , and also increased seed size	Na et al. (2018)
ω-3 long chain PUFAs, eicosapentaenoic acid (EPA) and docosahexaenoic acid (DHA)	Δ6-desaturase from *Ostreococcus. tauri* (OtΔ6), Δ6 fatty acid elongase from *Physcomitrella patens* (PSE1), Δ5-desaturase from *Thraustochytrium sp*. (TcΔ5), Δ12-desaturase from *Phytophthora sojae* (PsΔ12), ω3-desaturase from *Phytophthora infestans* (Piω3) and *Hyaloperonospora parasitica* (Hpω3), *O. tauri* Δ5 fatty acid elongase (OtElo5) and Δ4-desaturase gene from *Emiliania huxleyi* (EhΔ4)	Assembly of EPA and DHA pathway by seed-specific expression 5 genes (PSE1, TcΔ5, OtΔ6, Hpω3, and PsΔ12) and 7 genes (PSE1, TcΔ5, OtΔ6, Piω3, PsΔ12, OtElo5 and EhΔ4), respectively	EPA content was 16.2-17.2 % of total FAs in the 5-gene transgenics; EPA and DHA levels were 4.3-4.9% and 4.0% of total FAs in the 7-gene transgenics, respectively	Ruiz-Lopez et al. (2014); Usher et al. (2017)
ω-3 long chain PUFAs, EPA and eicosatetraenoic acid (ETA)	Δ9-elongase from *E. huxleyi* (EhElo9), Δ9-elongase from *I. galbana* (IsoElo9), Δ8-desaturase from *P. Salina* (PsΔ8), Δ5-desaturase from *E. huxleyi* (EhΔ5), ω3-desaturase from *P. infestans* (Piω3), and Δ12-desaturase from *P. sojae* (PsΔ12)	Assembly of Δ9-alternative pathway for ω-3 long chain PUFAs biosynthesis by seed-specific expression 6 genes (EhElo9, IsoElo9, PsΔ8, EhΔ5, Piω3 and PsΔ12)	Mean EPA levels in T3 seeds increased on average from 4.7 to 8.8%, with 16.9% of total FAs in the best line. Mean ETA content was 5.8-7.1%.	Ruiz-Lopez et al. (2015)
Medium-chain, saturated fatty acids (MCSFAs)	12:0-acyl-carrier thioesterase (UcFATB1) from *Umbellularia californica* and CsKASII	Seed-specific expression of UcFATB1 alone or together with CsKASII RNAi	Level of laurate (12:0) and myristate (14:0) were up to 40% of the seed oil in UcFATB1 transgenics; Level of 16:0 was increased from 7.5% up to 28.5% of total FAs in KASII-RNAi lines. Level of MCSFAs (12:0, 14:0 and 16:0) was up to 30% of the co-transformed seeds	Hu et al. (2017)
Medium-chain fatty acids (MCFAs; 8:0-14:0)	CpuFatB3 and CpuFatB4 from *C. pulcherrima*, CvFatB1 and CvFatB3 from *C. viscosissima*, CpFatB2 from *C. palustris*, UcFatB1from California bay, and ChFatB2 from *C. hookeriana;* a coconut lysophosphatidic acid acyltransferase (CnLPAT)	Seed-specific expression of each of those FatBs; Co-expression of two or three of those FatBs; Co-expression of FatB and CnLPAT	MCFAs were greatly accumulated in the individual FatB transgenics and much high levels occurred in coexpression lines	Kim et al. (2015)
Novel wax esters (WEs)	Acyl-ACP thioesterases (Thio), a fatty acid hydroxylase (FAH), *Marinobacter hydrocarbonoclasticus* WS (wax synthase), *Marinobacter aquaeolei* FAR (fatty acid reductase),	Co-expression of WS and FAR; Co-expression of WS, FAR and Thio or FAH	WEs with different compositions were produced in the transgenic seeds of FAR, WS and Thio; WEs with reduced chain lengths were produced in coexpression lines of MaFAR, MhWS, and Thio	Ruiz-Lopez et al. (2017)
Oleyl oleate wax esters (Oleyl oleate WEs)	*Marinobacter aquaeolei* FAR (Ma FAR) and *Simmondsia chinensis* WS (ScWS)	Co-expression of ScWS and MaFAR	Levels of oleyl oleate WEs were 21% of the seed oil TAGs in the coexpression lines of MaFAR and ScWS	Iven et al. (2016)
Nervonic acid (24:1Δ15)	β-ketoacyl-CoA synthase (LaKCS) from *Lunaria annua*, Arabidopsis β-ketoacyl-CoA reductase (AtKCR) and β-hydroxyacyl-CoA dehydratase (AtHCD)	Seed-specific expressing of LaKCS (*Vector1*); Co-expression of LaKCS, AtKCR and AtHCD (*Vector2*)	Nervonic acid in seed oil increased from null to 6-12% in *Vector1* transgenics; Nervonic acid level from *Vector2*- expressing line was significantly high in early seed development	Huai et al. (2015)
Seed oil	A patatin-related phospholipase AIIIδ (pPLAIIIδ) from Arabidopsis	Constitutive or seed-specific expression of pPLAIIIδ (*Vector1* or *Vector2*)	Seed oil increased greatly and cellulose reduced in *Vector1* transgenics; A increased seed oil content without negative effects in *Vector2* transgenics	Li et al. (2015)
ω-7 FAs (16:1Δ9, 18:1Δ11, 20:1Δ13)	A mutant Δ9-acyl-ACP (Δ9DES1), a 16:0-specific acyl-CoA desaturase (Δ9DES2), 3-keto-acyl-ACP synthase II (KASII) and FatB 16:0-ACP thioesterase (FatB)	Seed-specific co-expression of Δ9DES1and Δ9DES2 (*Vector1*); seed-specific suppression of CsKASII and CsFatB (*Vector2*)	ω-7 FAs was increased to 17% in *Vector1*-expression lines; ω-7 FAs was increased to 60-65% of total FAs in *Vector1* and *2* coexpression lines	Nguyen et al. (2015)
DHA	Δ;5-desaturase (Δ5D), Δ6-elongase (Δ5E), Δ6D, Δ6E, Δ4D, Δ12D , and ω3-desaturase (ω3D)	Overexpression of the 7 gens(Δ4D, Δ5D, Δ5E, Δ6D, Δ6E, Δ12D and ω3D)	levels of DHA was up to > 12% with very high ω3: ω6 ratios in transgenic seeds	Petrie et al. (2014)
Cuticular wax	Arabidopsis MYB96 (AtMYB96)	Overexpression of AtMYB96 driven by CaMV35S promoter	Significant increase of epicuticular wax crystals and total wax loads on the surfaces of transgenic leaves	Lee et al. (2014)

## Selected Examples Of Metabolic Engineering For Production Of The Designed Oils In *C. Sativa*

### Redesigning Acetyl Triacylglycerol (acetyl-TAG, acTAG) Synthesis for the Production of Superior Biodiesel and Lubricant Oils

AcTAGs (3-acetyl-1,2-diacyl-*sn*-glycerols) are unique and valuable triacylglycerols. Their molecular characteristics include two long-chain fatty acid acyl groups bound to the *sn*-1 and *sn*-2 positions of the glycerol molecule, respectively, and one acetyl group linked to the *sn*-3 position of the glycerol molecule (Durrett et al., 2010). Unlike ordinary TAGs (lcTAG) (three long-chain fatty acid acyl groups were bond on the three carbon atoms of the glycerol molecule, respectively), acTAGs have unique physical and chemical properties, showing their utility in a variety of applications. For example, their kinematic viscosity is 40% lower than that of ordinary TAGs. Therefore, acTAGs are excellent oils for use in the production of low-viscosity biofuels. The biodiesel produced can be used as a premium fuel for ships, trains, and generators. Another advantage of acTAGs is their low temperature tolerance. The biodiesel made from them is less prone to agglomeration and burning defects in low-temperature climates. AcTAGs can also be used to produce high-quality biodegradable lubricants (Liu et al., 2015a).

These high-value industrial oils cannot be synthesized in ordinary field oilseed crops, but in some wild plants, they are synthesized at high levels. For example, 98% of the seed oil of *Euonymus alatus* (burning bush) is acTAGs (Milcamps et al., 2005). Studies have shown that EaDAcT (*E. alatus* diacylglycerol acetyltransferase) catalyses the formation of acTAGs by the binding of a diacylglycerol (DAG) to an acetyl group at the *sn*-3 position. In the developing seeds of common oil crops, DGAT (diacylglycerol acyltransferase) or PDAT (phosphatidylcholine: diacylglycerol acyltransferase) catalyses DAG binding to a long-chain fatty acid acyl group to generate “regular” long-chain TAG (lcTAG) but does not catalyse the formation of acTAGs. The gene encoding EaDAcT was overexpressed in Camellia seeds, resulting in the seed oil containing up to 55% acTAGs. EaDAcT and DGAT or PDAT share the same substrate, DAG. If the activity of endogenous DGAT or PDAT can be silenced, more DAG can be used to generate acTAGs for EaDAcT. Silencing the expression of three *DGAT1* genes by RNAi suppression in *C. sativa* and overexpressing *EaDAcT* resulted in increasing acTAGs in the seed oil of transgenic *C. sativa* to 85%, and it was stably inherited (Liu et al., 2015a). Continuous field trials showed that compared with the wild-type, the seed weight and oil and protein content of this transgenic *C. sativa* were not significantly different, and the seed germination was normal. During seed germination, AcTAGs can also be degraded like regular lcTAGs for seedling growth (Liu et al., 2015b).

**Figure 1:**
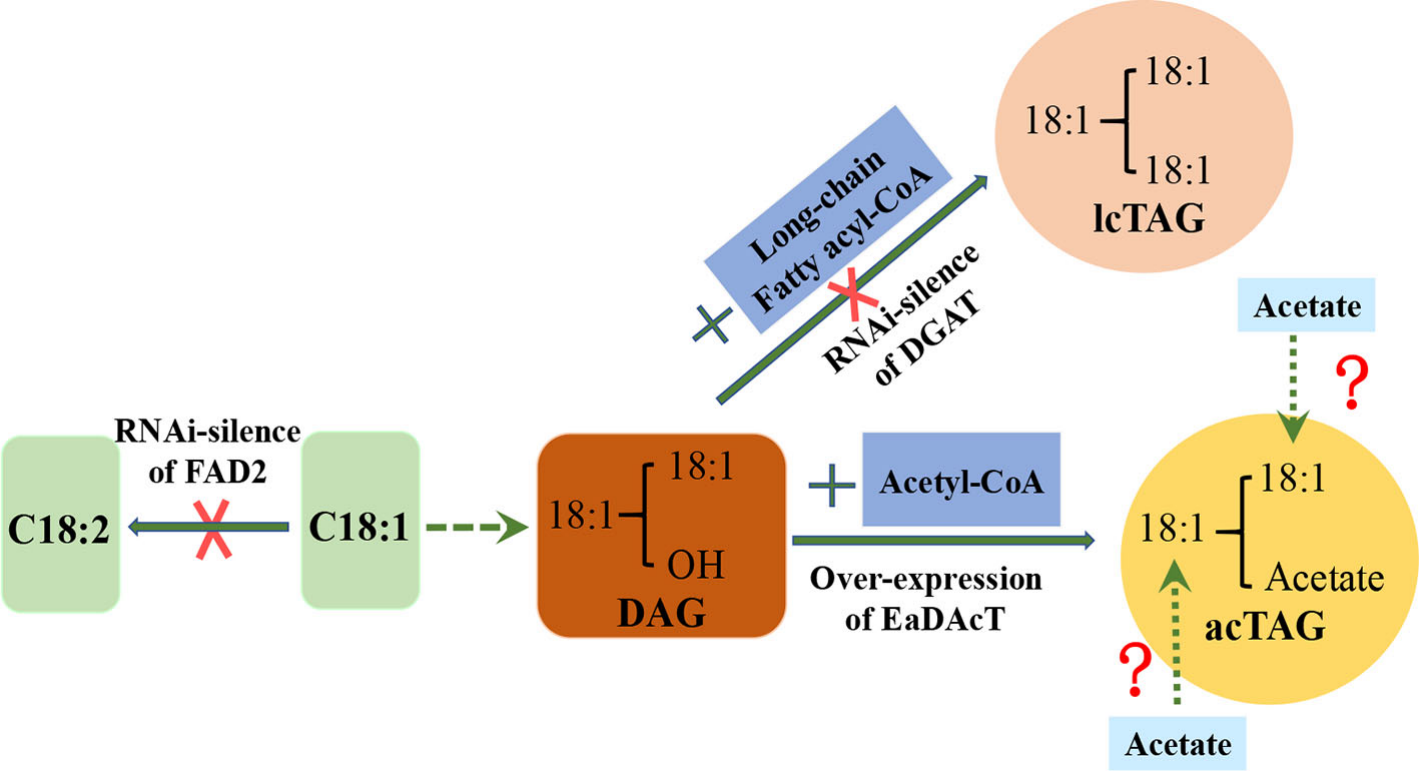
Strategy for redesigning biosynthesis of acetyl-TAG in *C. sativa* seeds. DAG, Diacylglycerol; lcTAG, Long-chain Triacylglycerol; acTAG, Acetyl Triacylglycerol; DGAT, Diacylglycerol acyltransferases; EaDAcT, *Euonymus alatus* diacylglycerol acetyl‐CoA transferase.

In order to further improve the physicochemical properties of acTAGs and expand their industrial applications, some studies have attempted to replace the long-chain polyunsaturated fatty acid acyl groups at the *sn*-1 and *sn*-2 positions of the acTAG molecule with other long-chain fatty acid acyl groups. The combination of monounsaturated oleic acid (18:1) acyl groups at the *sn*-1 and *sn*-2 positions of acTAGs improves the oxidation resistance of acTAGs. The oleic acid-rich strains of Camelina obtained by RNAi silencing of *GsFAD2* and were used as recipients, and vectors to over-express *EaDAcT* and RNAi-silence *CsDGAT* and *CsPDAT* were transferred to the receptor, resulting in an acTAG content in the seed oil of transgenic *C. sativa* as high as 70%, particularly, the content of 3-acetyl-1,2-dioleoyl-*sn*-glycerol was 47% (Liu et al., 2015a). Such oil showed a significant increase in the oxidation resistance. In addition, the middle chain fatty acid acyl group is bonded to the *sn*-1 and *sn*-2 positions of the acTAG molecule to further reduce the viscosity of the acTAG lipids (Durrett et al., 2010). This shows that changing the fatty acid acyl species at the *sn*-1 and *sn*-2 positions gives the acTAGs more desirable properties and broader industrial applications. Future efforts are needed to design a pathway to incorporate acetate into *sn-1* and *sn-2* positions of glycerol backbone (**Figure 1**).

Additionally, the introduction of acTAG into edible oilseed crops may provide an opportunity to develop reduced-calorie fats and oils with a molecular structure similar to that of existing commercial products such as SALATRIM (short and long acyl triglyceride molecule).

### Redesigning Hydroxylated Fatty Acid Biosynthesis for the Production of Oxidation Resistance Oils

The oxidation level of vegetable oils depends on the fatty acid composition. If the content of polyunsaturated fatty acid is high, the oxidizability of the vegetable oils is high. But high contents of saturated fatty acids, although more resistance to oxidation, reduce the fluidity of the oil, making it easily solidified. Vegetable oils containing high level of monounsaturated fatty acids (e.g. oleic acid,18:1Δ9) have high oxidation resistance and other good properties.

Camelina seed oil consists of about 45% polyunsaturated fatty acids, namely linoleic acid (18:2Δ9,12) and linolenic acid (18:1Δ9,12,15), while only 17% of seed oil is monounsaturated oleic acid (18:1Δ9). This seed oil containing high levels of polyunsaturated fatty acids is easily oxidized. RNAi technology was used to specifically silence the *FAD2* (Fatty acid desaturase 2), *FAD3* (Fatty acid desaturase 3), and *FAE1* (Fatty acid elongase 1) genes in camelina, obtaining a transgenic camelina seed oil with significantly reduced content of polyunsaturated fatty acids and a high level of oleic acid (from 18% in wild-type to 65% in the transgenic seeds). Such camelina oil exhibited significantly improved oxidation resistance (Cahoon et al., 2007). In addition, another strategy is to express an enzyme that catalyses the formation of monounsaturated hydroxylated fatty acids in the seeds of camelina, promoting the biosynthesis and accumulation of high levels of hydroxylated fatty acids. Because this fatty acid is extremely resistant to oxidation, the inoxidizability of the seed oil can be increased. Hydroxylated fatty acids have been widely used in the industrial production of resins, waxes, nylons, plastics, lubricants, and cosmetics.

Castor bean (*Ricinus communis*) seed oil consist of up to 90% ricinoleic acid (18:1Δ9,12OH) (a type of monounsaturated hydroxylated fatty acid, HFA). A fatty acid hydroxylase FAH12 was found to catalyze the oleic acid molecule (18:1Δ9) bound to PC to generate a hydroxyl group at the Δ12 carbon atom. Seed-specific expression of the *RcFAH12* gene from *R. communis* resulted in ricinoleic acid in camelina seeds reaching up to 6% (Lu and Kang, 2008). Correspondingly, the oxidation resistance is significantly higher than that of non-transgenic camelina oil. Lesquerelic acid is another hydroxylated fatty acid similar to ricinoleic acid and accumulates in high levels in plant seeds of the *Physaria* genus and *Cruciferae* family. A fatty acid condensing enzyme, LfKCS derived from *Physaria fendleri*, specifically catalyses the elongation of ricinoleic acid to hydroxyalkanoic acid. The synergistic expression of *RcFAH12* and *LfKCS* in camelina seeds not only increased ricinoleic acid from 14% to 19% but also resulted in hydroxyarsenoic acid reaching 8%. The total amount of hydroxylated fatty acids reached 27%. The oxidation resistance of this camelina oil was greatly improved, and seed vigor was not affected (Snapp et al., 2014). Clearly, LfKCS expression accelerates the removal of hydroxylated fatty acids from the phosphatidylcholine (PC) synthesis pool into the acyl-CoA pool to finally form TAGs. A phospholipase C-like protein (RcPLCL1) from castor bean was identified to have hydrolyzing activities on both PC and phosphatidylinositol (PI) substrates (Aryal and Lu, 2018). Co-expression of *RcPLCL1* and *RcFAH12* resulted in accumulation of HFAs up to 24% of total FAs in *C. sativa* seeds (Aryal and Lu, 2018) with less detrimental effect on seed germination, showing that RcPLCL1 can promote the transfer of RcFAH12-formed HFAs on PC into DAG to generate TAGs containing HFAs.

With the Arabidopsis model plant as a receptor, the co-expression of *R. communis RcFAH12* with *RcDGAT2* and *RcPDAT* which controls the final acylation reaction of TAG synthesis can further increase the hydroxylated fatty acid content in seed oil up to 29% (Burgal et al., 2008; van Erp et al., 2011). Co-expression of *RcFAH12* and *RcPDCT* (phosphatidylcholine: diacylglycerol choline phosphotransferase) increased the HFA accumulation in Arabidopsis seeds from approximately 10% to 20% (Hu et al., 2012). Unlike conventional DGAT, PDAT, and PDCT, R. communis homologs are specific for hydroxylated fatty acid substrates. These castor enzymes can accelerate the transfer of HFAs from the PC pool and CoA pool into DAG to form hydroxylated TAGs. It is hypothesized that the co-expression of these three enzyme genes with *RcFAH12* in camelina seed will allow the amount of hydroxylated TAG to accumulate to levels appropriate for commercial use. Since fatty acid thioesterase A (FatA) and fatty acid thioesterase B (FatB) in camelina plastid were identified to be specific for oleoyl-ACP and palmitoleic acid-ACP, respectively (Rodriguez-Rodriguez et al., 2014), in the future, the molecular manipulation of these two enzymes as targets will allow cells to selectively accumulate oleic acid (18:1Δ9) or palmitoleic acid (16:1Δ9) in TAGs and increase the resistance to oxidation of camelina seed oil.

### Assembling Medium-Chain Fatty Acid Biosynthesis for the Production of High-Quality Jet Oils

Jet fuels (Jet A and Jet-A1 fuels) consist mainly of C8-C16 alkanes and aromatic hydrocarbons (Kallio et al., 2014). The main fatty acids of most common oilseeds, such as camelina, are 18C fatty acids, which are directly used to process aviation fuels with poor quality and lengthy processes. Vegetable oils rich in caprylic acid (8:0), capric acid (10:0), lauric acid (12:0), myristic acid (14:0) are excellent resources for the production of aviation biofuels (Dyer et al., 2008). Medium-chain fatty acids (MCFAs) are also widely used in the production of detergents, soaps, cosmetics, surfactants, and lubricants.

The palm kernel of the tropical palm plant (*Elaeis guineensis* Jacq.) and the coconut meat of coconut (*Cocos nucifera* L.) are rich in lauric acid (46% to 52%) and decanoic acid (16% to 19%), serving as the main sources of commercial MCFAs. Some of the Lythraceae Cuphea plants can be enriched with >90% of MCFAs. For example, the seed of *Cuphea viscosissima* contains about 25% caprylic acid and about 64% capric acid. The seed of *Cuphea pulcherrima* is enriched with about 95% caprylic acid (Knothe, 2014). These plants have indeterminate agronomic traits such as infinity of inflorescence, shattering, and seed dormancy, being difficult to use in the commercial production of seed oil. However, they can be used as an excellent gene source for MCFA biosynthesis (Filichkin et al., 2006).

**Figure 2:**
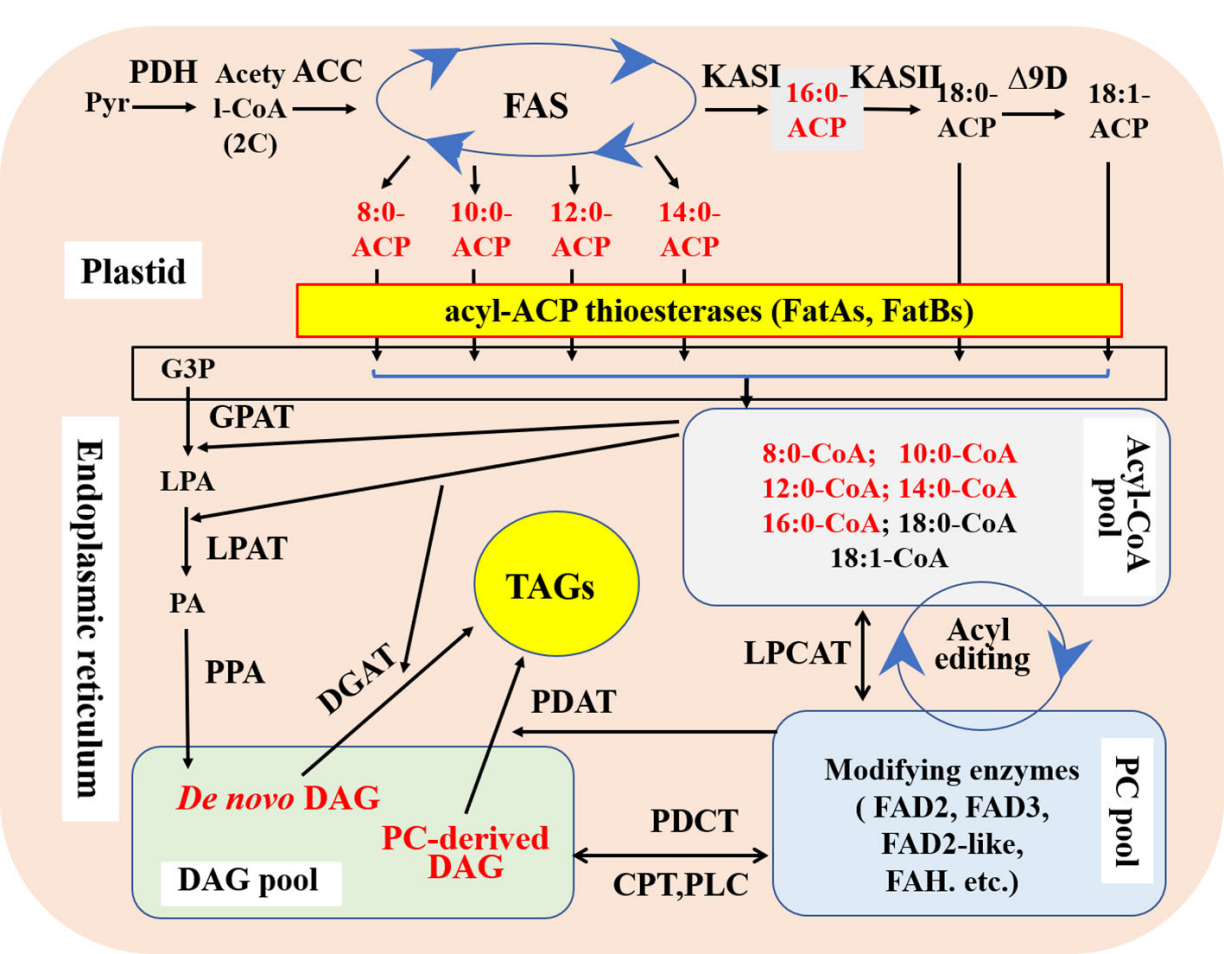
Schematic diagram showing the key steps of *de novo* synthesis of fatty acids and triacylgltcerols with main modified targets for medium-chain fatty acid production in plant seeds. FAS; Fatty acid synthase; FatA, Fatty acid thioesterase A; FatB, Fatty acid thioesterase B; KASII, β‐ketoacyl‐ACP synthase II; DAG, Diacylglycerol; DGAT, Diacylglycerol acyltransferases; TAG, Triacylglycerol; PDAT, Phospholipid diacylglycerol acyltransferase; GPAT, glycerol-3-phosphate acyltransferase; LPAT, Lysophosphatidic acid acyltransferase; PDH, Pyruvate dehydrogenase; ACC, Acetyl-CoA carboxylase; Pyr, Pyruvic acid.

The *de novo* synthesis of plant fatty acids occurs in the plastids. Acetyl-CoA and malonyl-ACP are catalyzed by β-ketoacyl-ACP synthase III (KASIII) to form 4C β-ketoacyl-ACP. Then, under the action of the fatty acid synthase (FAS) complex, each cycle adds 2 carbon atoms until 16-carbon palmitate-ACP (16:0-ACP) is formed. 16:0-ACP can be further extended to stearic acid-ACP (18:0-ACP) under the action of KASII. It is also possible to dissociate palmitate from ACP and terminate the elongation of the fatty acid carbon chain under the catalysis of acyl-ACP thioesterase FatB. The 18C fatty acids synthesized in the plastids, namely stearic acid (18:0) and oleic acid (18:1), are catalyzed by FatA thioesterase to dissociate from ACP (**Figure 2**). Therefore, acyl-ACP thioesterases are the major determinants of the synthesis of fatty acid carbon chain lengths in the plastids (Li-Beisson et al., 2013).

After being transferred from the plastids, MCFAs and palmitic acid (16:0) bind to CoA and then enter the endoplasmic reticulum of the cytoplasm. After a series of reactions, they eventually bind to the *sn*-1, 2, and 3 positions of the glycerol carbon skeleton and turn into TAG. Glycerol-3-phosphate acyltransferase (GPAT), lysophosphatidic acid acyltransferase (LPAT), and DGAT in turn catalyze the esterification of MCFAs-CoA and 16:0-CoA into TAG molecules, and DGAT is thus the key enzyme to accumulate high levels of MCFAs and palmitic acid (Lu et al., 2011). It has been found that LPAT in most oil crops has substrate selectivity for unsaturated fatty acid-CoA (such as oleic acid-CoA) and no selectivity for MCFAs-CoA (Nlandu Mputu et al., 2009). CnLPAT derived from *Cocos nucifera* has a strong substrate specificity for lauric acid (12:0)-CoA (Kim et al., 2015).

To date, genes encoding FatB enzymes with higher substrate specificity for MCFAs than for palmitoyl-ACP has been isolated from *Cuphea* and *Umbellularia californica* seeds containing high levels of MCFAs. Heterologous overexpression of these *FatBs* resulted in the synthetic accumulation of MCFAs in *Brassica napus* and *Arabidopsis* seeds (Tjellstrom et al., 2013). RNAi was used to silence *KAS*II to block the production of 18:0-ACP from 16:0-ACP, obtaining transgenic seeds that accumulated high levels of 16:0-ACP (Pidkowich et al., 2007). The transgenic camelina seeds specifically expresses the *CpFatB2* gene derived from *C. palustris* accumulated 25% myristic acid (14:0). The *C. sativa* seed respectively accumulated lauric acid (12:0) to 18%, and capric acid (10:0) to 10% following the overexpression of *UcFatB1*, and *ChFatB2* (*C. hookeriana FatB2*), respectively. The *UcFatB1* gene from *U. californica* and the *CnLPAT* gene from coconut were co-expressed in camelina seeds, resulting in up to 30% accumulation of lauric acid (12:0) in camelina seeds (Collins-Silva et al., 2011).

Kim et al. (2015) performed the functional identification of FatBs from the *C. viscosissima* and *C. pulcherrima*, and subsequently used these FatBs for assembly of MCFA synthesis pathway in camelina seeds. Transcriptome analysis revealed that three FatB cDNAs, namely *CpuFatB3*, *CvFatB1*, and *CpuFatB4*, were abundantly expressed in developing seeds, showing positive association with the accumulation of MCFAs.

*CpuFatB4* is selective for 12:0-ACP, 14:0-ACP, and 16:0-ACP. The content of palmitic acid (16:0) in camelina seeds overexpressing *CpuFatB4* rose to 43.5% (5 times higher than in the wild-type), and the content of myristic acid (14:0) reached 8%. Similar to CpuFatB4, CpuFatB3 has broad-spectrum substrate specificity for various MCFA-ACPs. The accumulation of capric acid (10:0) in the seeds of *C. sativa* overexpressing *CpuFatB3* reached 1.2%, while only small amounts of other MCFAs (8:0, 12:0, 14:0) were synthesized. The accumulation of capric acid and palmitic acid in camelina seeds overexpressing *CvFatB1* reached 9% and 16%, respectively, and the contents of other MCFAs (8:0, 12:0, 14:0) were also low. Two or three genes encoding FatB enzymes were co-expressed, resulting in the accumulation of various MCFAs such as C8-C16 in transgenic *C. sativa* seeds, but each fatty acid content was lower than the corresponding fatty acid content in the seeds with only one gene for FatB enzyme. Further over-expression of *CpFatB2* or *UcFatB1* and coconut *CnLPAT* in camelina seeds resulted in the synthesis of more MCFAs. More importantly, co-expressing MCFA-specific FatB and CnLPAT not only increased the MCFA content but also had no negative effect on the total oil content. The above-mentioned metabolically modified engineered camelina strains enriched with MCFAs can be directly used for the production of high-quality jet fuel that is highly resistant to low temperatures.

### Assembling ω-3 Long-Chain Polyunsaturated Fatty Acid Biosynthesis for the Production of Oils With Healthcare Applications

**Figure 3:**
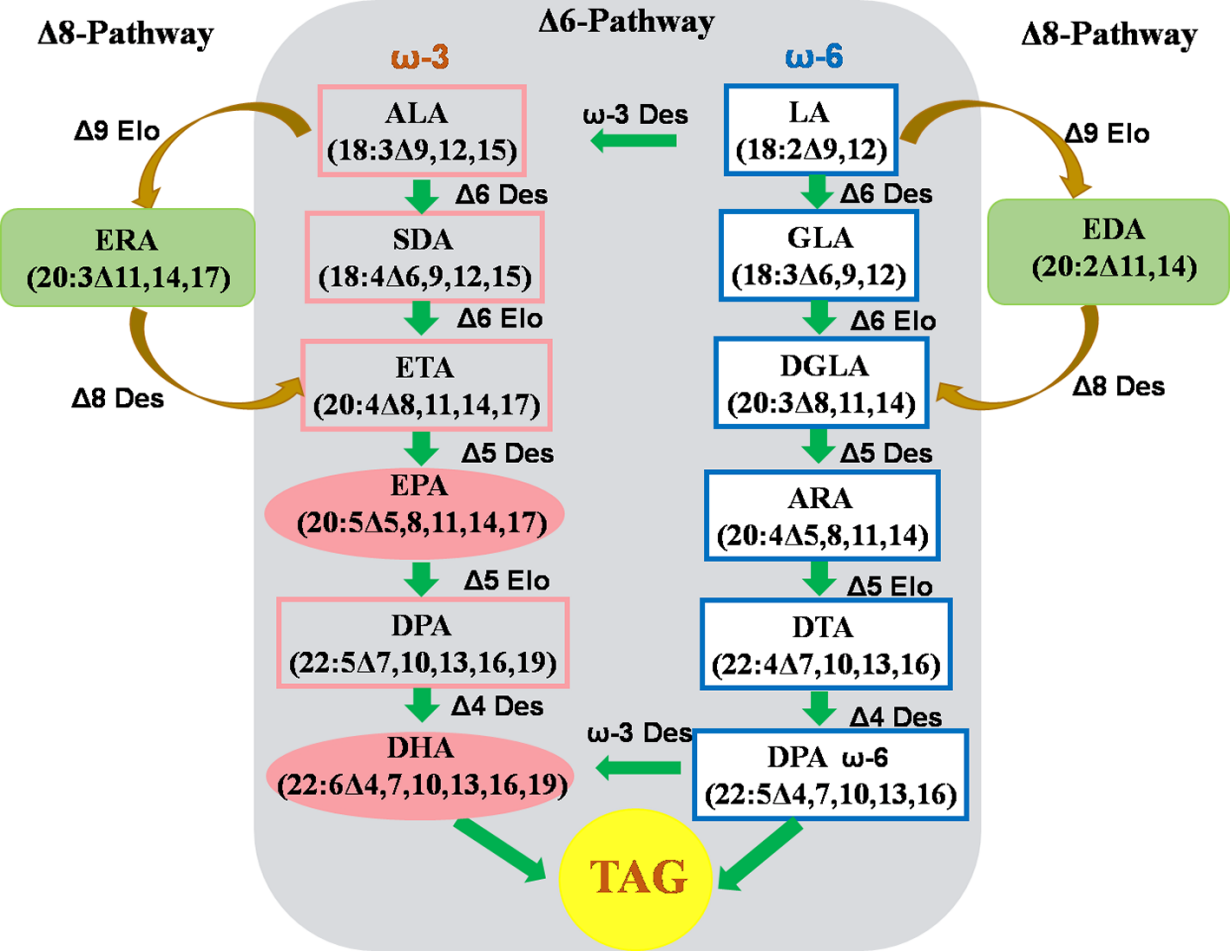
Strategy to assembly biosynthesis of ω-3 LC-PUFAs in *C. sativa* seeds. Δ3 Des, Δ3-desaturase; Δ4 Des, Δ4-desaturase; Δ5 Des, Δ5-desaturase; Δ6 Des, Δ6 -desaturase; Δ8 Des, Δ8-desaturase; Δ5 Elo, Δ5-elongase; Δ6 Elo, Δ6-elongase; Δ9 Elo, Δ9-elongase; ALA,α-Linolenic acid; ARA, Arachidonic acid; DGLA, Dihomo-γ-linolenic acid; DHA, Docosahexaenoic acid; DPA, Docosapentaenoic acid; DTA, Adrenic acid; EDA, Eicosadienoic acid; EPA, Eicosapentaenoic acid; ERA, Eicosatrienoic acid; ETA, Eicosatetraenoic acid; GLA, γ-Linolenic acid; LA, Linoleic acid; SDA, Stearidonic acid; TAG, Triacylglycerol. (The figure is modified according to Ruiz-Lopez et al., 2015.)

Omega-3 fatty acids (ω-3 FAs) are fatty acids that have one double bond at the 3rd carbon atom of the methyl terminal of the carbon chain, including a-linolenic acid (ALA,18:3Δ9,12,15), eicosapentaenoic acid (EPA,20:5Δ5,8,11,14,17), and docosahexaenoic acid (DHA, 22:6Δ4,7,10,13,16,19). In particular, long-chain polyunsaturated fatty acids (ω-3-LC-PUFAs), such as EPA and DHA, derived from fish oil are extremely important for human health, dietary nutrition, and brain development. In order to establish a renewable resource that can replace fish oil for the production of EPA and DHA, many studies have been devoted to the assembly of the EPA and DHA biosynthetic pathways in the developing seeds of common oil crops in order to achieve the “factory” production of EPA and DHA to meet growing market demand (Ruiz-Lopez et al., 2014; Betancor et al., 2015; Malik et al., 2015; Ruiz-Lopez et al., 2015). The EPA and DHA pathways have been assembled in the seeds of oil crops such as soybeans. However, the accumulation of EPA and DHA is low, and it is difficult to commercialize. Camelina seed contains >30% ALA, which is the starting substrate required for the synthesis of EPA and DHA, making it a good platform for assembling the ω-3-LC-PUFA synthesis pathway (**Figure 3**).

There are two ways for biosynthesis of ω-3-LC-PUFA: the conventional Δ6 pathway and the unconventional Δ8 pathway (Ruiz-Lopez et al., 2015). The Δ6 pathway begins with ALA. The synthesis of EPA requires one Δ6 and Δ5 desaturase (Δ6Des and Δ5Des) and one ELO-type Δ6 carbon chain elongase (Δ6Elo); the further synthesis of DHA requires catalysis by one ELO-type Δ5 carbon chain elongase (Δ5Elo) and one Δ4 desaturase (Δ4Des).

The seed-specific coexpression vectors for five genes were constructed and introduced into camelina to assemble the conventional Δ6 pathway of EPA synthesis in developing seeds. The genes used include OtΔ6 desaturase from the eukaryotic microalgae *Ostreococcus tauri*, TcΔ5 desaturase from marine fungus *Thraustochytrium* sp, Piω-3 desaturase from *Phytophthora infestans*, PsΔ12 desaturase from *Phytophthora sojae*, and Δ6 fatty acid carbon chain elongation enzyme PSE1 from *Physcomitrella patens*. The co-expression of these five genes led to 31% EPA accumulation in camelina oil (average EPA 24%) (Ruiz-Lopez et al., 2014). EhΔ4 desaturase from *Emiliania huxleyi* and Δ5 elongase OtElo5 from eukaryotic microalgae were then inserted with these five gene vectors to construct seven gene coexpression vectors. The co-expression of these seven genes in the seed of *C. sativa* resulted in the accumulation of up to 14% DHA (average 8%) (Ruiz-Lopez et al., 2014). The contents of EPA and DHA in this transgenic camelina reached the levels in fish oil, representing the highest EPA and DHA accumulation levels to date have been successfully obtained by assembling biosynthetic pathways in commercial oil crop seeds. More importantly, there is no accumulation of other harmful intermediate metabolites in the seed oil of camelina with these complete EPA and DHA synthetic pathways. The accumulation of EPA and DHA does not negatively affect other agronomic traits, and thus the transgenic plants exhibit normal growth and development. This new camelina germplasm obtained by such metabolic engineering can serve as a high-quality renewable resource for EPA and DHA, and it will be further developed in the future for the commercial production of a series of medicines and nutraceuticals rich in the long-chain ω-3 fatty acids EPA and DHA.

The Δ8 pathway of the unconventional synthesis of EPA is essentially a by-pass pathway, found in some microalgae (e.g. *Pavlova salina* and *Isochrysis galbana*) cells (Ruiz-Lopez et al., 2015). The starting substrate for the Δ8 pathway is also a-linolenic acid (ALA). ALA is elongated two carbon atoms by the ELO-typeΔ9 carbon chain elongase (Δ9Elo) to produce eicosatrienoic acid (20:3Δ11,14,17; ω-3, ERA). Next, Δ8 desaturase catalyses ERA to form arachidonic acid (20:4Δ8,11,14,17; ω-3, ETA). Finally, ETA is catalytically converted to EPA by Δ5 desaturase (20:5Δ5,8,11,14,17; ω-3). The Δ8 pathway has now been successfully assembled and expressed in the developing seeds of camelina, and the accumulation of long-chain ω-3 fatty acids (EPA and ETA) has reached levels up to 26.4% in transgenic *C. sativa* seed oil (Ruiz-Lopez et al., 2015). In addition, linoleic acid (18:2Δ9, 12, LA) is also the substrate for the elongase Δ9Elo. Δ9Elo catalyzes the addition of two carbon atoms to LA to extend it to eicosenoic acid (20:2Δ11,14; ω-6, EDA). Under the action of Δ8 desaturase, EDA is converted to eicosatrienoic acid (20:3Δ8,11,14; ω-6, DGLA). Finally, the Δ5 desaturase catalyzes the conversion of DGLA into arachidonic acid (20:4Δ5,8,11,14; ω-6, ARA), which in turn is acted on by Δ5Elo, Δ4Des, and ω-3Des to form DHA. Camelina expressing the Δ8 pathway also requires a precise metabolic modification to reduce the synthesis of the two ω-6 fatty acids DGLA and ARA and promote synthesis to accumulate more EPA and DHA.

For the plant-based production of ω-3 LC-PUFAs, two reviews (Napier et al., 2015; Haslam et al., 2016) described several examples of “proof-of-concept” and progresses in developing transgenic plants enriched ω-3 fish oils. Although promising achievements were obtained in recent years, the metabolic bottleneck remains to be resolved, such as low levels of these non-native fatty acids accumulated in the transgenic seeds and less knowledge on interaction between the introduced pathway and the endogenous metabolic network. With the success of the field testing of ω-3 LC-PUFAs-enriched camelina, large-scale production of the novel oil by plants will provide an alternative, sustainable source of ω-3 fish oils for increasing end-users.

### Redesigning ω-7 Monounsaturated Fatty Acid Biosynthesis for the Production of High-Valued-Added Oils

Omega-7 fatty acids (ω-7 FAs), including palmitoleic acid (16:1Δ9), 11-octadecenoic acid (18:1Δ11) and 13-eicosenoic acid (20:1Δ13), have important industrial, nutritional, and pharmaceutical values. ω-7 FAs are also the best fatty acids for producing high-quality biodiesel (Durrett et al., 2010). These rare fatty acids are mostly synthesized in the seeds of some wild plants such as cat's claw (*Dolichandra unguis-cati*, 64% 16:1Δ9 and 15% 18:1Δ11) and sea buckthorn (*Hippophae rhamnoides*, 32% 16:1Δ9). Because of the poor agronomic traits of these nonergonomic plants, they have not been commercialized to date. Seeds of common oil crops accumulate very small amounts of ω-7 FAs. Metabolic engineering techniques could be employed to assemble ω-7 FA synthesis pathways in common oilseed crops in order to achieve the commercial production of ω-7 FA-rich vegetable oils (Wu et al., 2012; Nguyen et al., 2015). To assemble this pathway in soybean, our laboratory constructed a seed-specific expression vector for co-expression of the Δ9 desaturase gene that catalyzes palmitic acid (16:0) to palmitoleic acid (16:1Δ9) and the gene encoding a DGAT enzyme. The content of ω-7 FAs in the transgenic seeds with the co-expression of the two genes was greater than 29%. With this soybean oil, a series of functional foods rich in ω-7 FAs can be produced. In addition, in order to develop a new-type of tobacco specifically used for biofuel production, our laboratory has also assembled ω-7 FA biosynthetic pathways in tobacco vegetative organs. The transgenic tobacco leaves accumulated high levels of ω-7 FAs (Xue et al., 2013).

**Figure 4:**
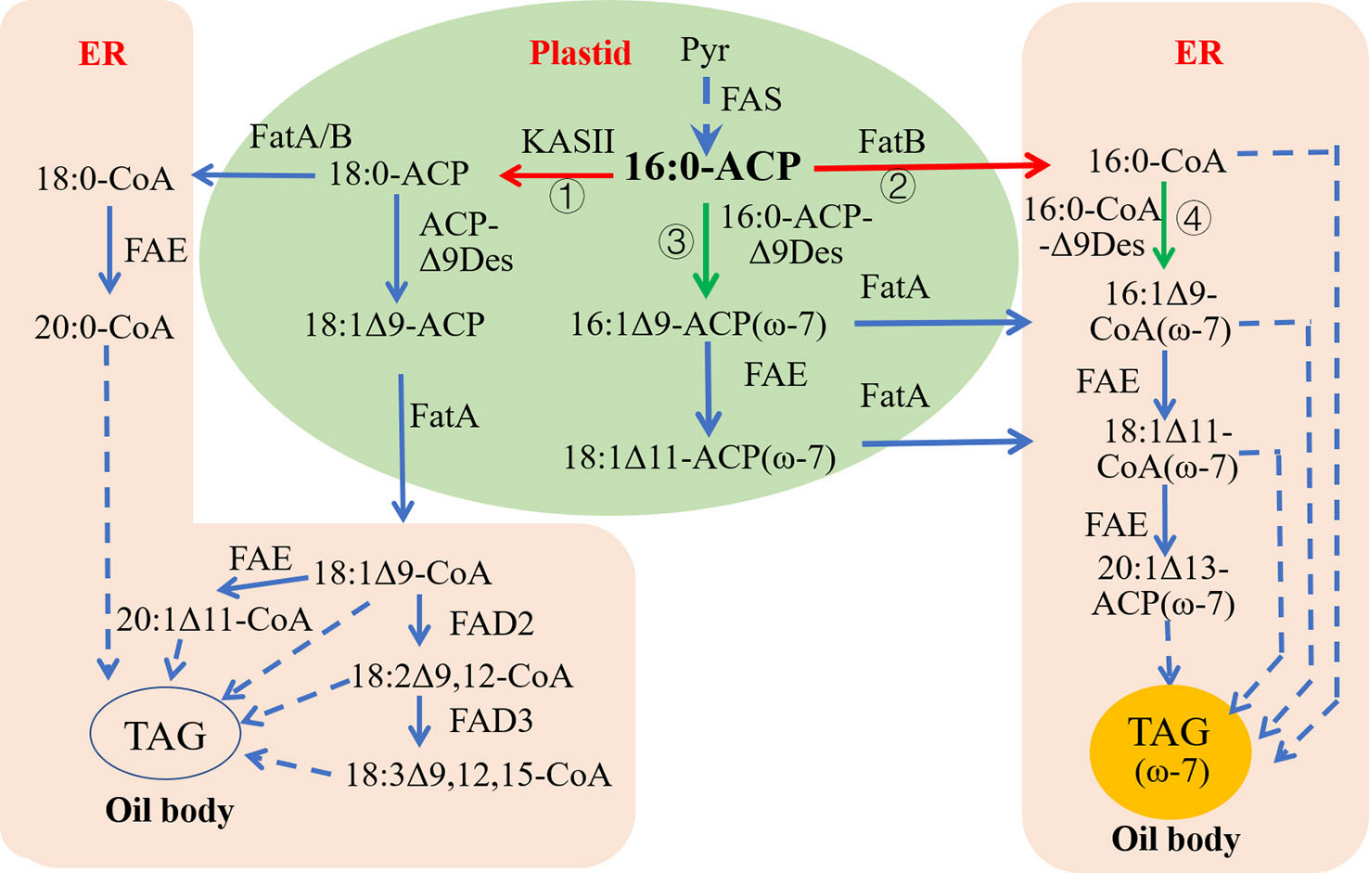
Strategy redesigning biosynthetic pathways for ω-7 FAs production in common oil seeds. ER, Endoplasmic reticulum; FAS, Fatty acid synthase; FatA, Fatty acid thioesterase A; FatB, Fatty acid thioesterase B; FAE, Fatty acid elongase; KASII, β‐ketoacyl‐ACP synthase II; TAG, Triacylglycerol. Green arrows indicate up-regulation, red arrows indicate down-regulation; ① and ② are to silence KASII and FatB, respectively, to increase pool sizes of 16:0-ACP in plastid to provide more substrates fo16:0-ACP Δ9-desaturase; ③ is to introduce a plastid-localized acyl-ACP Δ9-desaturase with high 16:0-ACP activity; ④ is to introduce an ER-localized 16:0-CoA-specific Δ9-desaturase to catalyze 16:0-CoA into 16:1-CoA in cytoplasm.

The starting substrate for the synthesis of ω-7 FAs like palmitoleic acid is palmitoyl (16:0)-ACP, which is synthesized in the plastid. There are two downstream enzymatic reaction pathways following the 16:0-ACP generated in the plastid of common oil crops. In the first pathway, under the action of KASII, 16:0-ACP plus two carbon atoms are extended to produce stearoyl (18:0)-ACP. 18:0-ACP can be directly transferred from the plastid into the cytoplasmic endoplasmic reticulum as 18:0-CoA. 18:0-ACP can also be catalyzed by the acyl-ACP-Δ9 desaturase (acyl-ACP-Δ9Des) in the plastid to generate monounsaturated oleoyl (18:1Δ9)-ACP, which is then transferred from the plastid and bound to CoA (18:1Δ9-CoA) to enter the cytoplasmic endoplasmic reticulum. In the second pathway, 16:0-ACP is dissociated from ACP by the action of FatB, and palmitic acid (16:0) is transferred from the plastid and bound to CoA to enter the cytoplasmic endoplasmic reticulum as 16:0-CoA. In some wild plant seeds rich in ω-7 fatty acids, 16:0-ACP is first converted to palmitoleoyl (16:1Δ9)-ACP (ω-7) in the plastid under the action of acyl-ACP-Δ9Des with substrate specificity for 16:0-ACP, followed by the action of the fatty acid elongate (FAE) to produce 11-octadecenoyl (18:1Δ11)-ACP (ω-7). Finally, under the influence of FatA, 16:1Δ9 and 18:1Δ11 are dissociated from ACPs and transferred out of the plastid and bound to CoA to enter the cytoplasmic endoplasmic reticulum as 16:1Δ9-CoA and 18:1Δ11-CoA, respectively (**Figure 4**).

Currently, a successful strategy for assembling ω-7 FA biosynthesis in *C. sativa* seeds involves expressing an acyl-ACP-Δ9Des that specifically catalyzes 16:0-ACP to produce 16:1Δ9-ACP in the plastids to increase plastid 16:1 Δ9-ACP synthesis. The simultaneous expression of one or two acyl-CoA-Δ9 desaturase (acyl-CoA-Δ9Des) capable of specifically catalyzing 16:0-CoA produced in the cytoplasmic endoplasmic reticulum to form 16:1Δ9-CoA will further increase the accumulation of 16:1Δ9 and its elongation products (18:1Δ11 and 20:1Δ13) in the transgenic camelina seeds.

Nguyen et al. (2015) applied this strategy to assemble the ω-7 FA biosynthetic pathway in camelina and obtained an engineered strain that accumulated high levels of ω-7 FAs. The seed-specific co-expression of one acyl-ACP-Δ9Des with strong specificity for 16:0-ACP and two acyl-CoA-Δ9Des with strong specificity for 16:0-CoA resulted in an increase in the accumulation of ω-7 FAs in the seeds of transgenic camelina from 0.6% in the wild-type to 17%. In this transgenic camelina, resynchronizing the RNAi silencing of endogenous *CsKASII* and *CsFatB* further increased the ω-7 FA content to an average of 60% of total lipids, and the saturated fatty acid content was correspondingly reduced from 12% in wild-type to 5% in transgenic seeds. This engineered camelina line with high accumulation of ω-7 FAs did not exhibit poor agronomic traits. Seed weight, protein content, and oil content did not differ from those in the control, and seed germination and seedling development were normal. Camelina oil rich in ω-7 fatty acid can be used to more efficiently produce various medicines and functional health products and foods. Physicochemical tests have shown that the thermodynamic properties of these camelina oils rich in ω-7 FAs are significantly improved compared to those of wild type camelina oils (Nguyen et al., 2015). The ω-7 FA-enriched oils can be used to produce high-value-added industrial products such as high-value biodiesel and polyurethanes.

### CRISPR/Case9 Gene Editing Technology in Designer Oil Production

The CRISPR/Cas9 gene editing has evolved as the most powerful tool for efficient and specific genome engineering to create genetic models for both fundamental research and crop genetic improvements although this new technology is being developed rapidly (Shan et al., 2013; Van Campenhout et al., 2019; Wolter et al., 2019). This gene editing system has been successfully used to generate a broad range of stable mutagenesis at specific locus in several important crops such as rice (Zhang et al., 2014), wheat (Wang et al., 2019), maize (Svitashev et al., 2015), soybean (Li et al., 2015), tomato (Ito and Nakano, 2015), potato (Butler et al., 2015), barley and *Brassica oleracea* (Lawrenson et al., 2015). The CRISPR-Cas9 system can also be employed to achieve simultaneous editing multiple loci by expressing different sgRNAs targeting the genes (Lowder et al., 2015; Ma et al., 2015). Many agronomic traits of crops were modified by CRISPR-Cas9, including yield and quality-related traits, plant nutrition and development, resistances to various stresses, and domestication (Chen et al., 2019). Notably, simultaneous targeting genes related to meiosis and fertilization by this technology produced a system enabling the clonal reproduction from F1 hybrid rice, thus stably preserving the favorable high degree of heterozygosity (Wang et al., 2019).

As highlighted by recent studies, CRISPR-Cas9 system was successfully used in *C. sativa* for seed oil quality improvement (Jiang et al., 2017; Morineau et al., 2017; Ozseyhan et al., 2018). The allohexaploid camelina contains three closely-related sub-genomes with each gene having three pair of alleles. Camelina seed oil is dominated by polyunsaturated fatty acids (linoleic and linolenic acids) and the development of new varieties rich in monounsaturated fatty acids (particularly oleic acid) is desirable. By targeting all three homeologs of the *CsFAD2* gene, Jiang et al. (2017) obtained a diverse set of genetic combinations with single, double and triple knockouts. In these mutant lines, oleic acid content was increased from 16% to over 50% of total fatty acids, whereas polyunsaturated fatty acids were significantly reduced. Similarly, Morineau et al. (2017) also obtained selectively targeted mutagenesis of *CsFAD2* genes by CRISPR-Cas9, showing the mutants with decreased polyunsaturated fatty acids and concomitant increase of oleic acid in the oil. Another lipid-related gene, *FAE1*(fatty acid elongase1), was effectively deactivated in camelina by CRISPR-Cas9 targeting three *FAE1* alleles simultaneously (Ozseyhan et al., 2018). Homozygous knockout mutants without growth defect were generated in a single generation by an egg cell-specific Cas9 expression, exhibiting that content of C20-C24 very long-chain fatty acids are reduced to less than 2% of total fatty acid compared to over 22% in the wild type. However, knocking out FAE1 increased levels of oleic acid or α-linolenic acid in camelina oils, which are desirable for industrial or food/feed utilizations. The different allelic combinations generated by the gene editing above allowed an unbiased characterization of gene dosage and function in this allohexaploid species, providing a valuable resource of genetic variability for precise plant breeding. It is promising that CRISPR-Cas9 technology will be used to effectively mutate other oil/fatty acid-related genes in camelina for genetic fine-tunning of oil trait.

## Future Prospects

Arabidopsis has long been used as a model plant although it is in fact a “wild grass”, not a cultivated agricultural plant. For comprehensive understanding of the complexity of many commercial crops, it is necessary to develop a model crop. In this regard, rice (*Oryza sativa*) is recognized as the model plant for cereal crops and species in Gramineae. Similarly, *Camelina sativa* has emerged as a new model plant for agricultural plants, especially for oil crops. Due to its short life cycle and an efficient genetic transformation like Arabidopsis, camelina is also developed as a “platform crop” with its seeds as “bioreactors” for commercial production of many high-valued products, followed by a new round of camelina commercial planting and a research boom on camelina-based industry worldwide.

As described above, several strategies are available to design high-valued fatty acids/oils by metabolic engineering in camelina seeds. These tools include optimizing enzymatic activity of the lipid-related enzymes, modifying a key gene, and/or coordinately expressing multiple genes associated with fatty acid synthesis and oil accumulation. Future achievements in this field require to obtain more knowledge on the key genes in lipid synthesis and accumulation, the networks responsible for regulation of metabolic carbon partitioning and target lipid synthesis, the rational redesign of novel enzymatic activities to synthesize the target FAs, and the effective assembly of lipid pathways consisting of multiple genes in the host plant.

Genome-wide association studies (GWAS) and multiple “omics” tools including genome resequencing, transcriptomics, and metabolomics are increasingly used to identify new genes involved in FA synthesis and modification and their incorporation into TAGs. For example, a GWAS using 391 wild Arabidopsis accessions revealed four to nineteen genomic regions responsible for FA composition in seed oil, showing that the most regions identified contained candidate genes that had never before been implicated in lipid metabolism (Branham et al., 2015). This tool was also used to examine a number of loci containing candidate genes involved in wax biosynthesis in camelina (Luo et al., 2019). By mining sequence data, the candidate enzymes for target FAs were identified, and subsequently used to manipulate *C. sativa* oil composition towards a superior biofuel and bio-based lubricant oil (Kim et al., 2015; Nguyen et al., 2015).

To enrich the designed FAs/oils in the host, systems biology approaches are needed to obtain a greater understanding of the networks responsible for metabolic carbon partitioning and the transcription factors regulating the related metabolism. More promising, synthetic biology tools like GoldenBraid 2.0 that enable interchangeable, modular assembly of transgene cassettes (Sarrion-Perdigones et al., 2013) can also be employed to effectively introduce the complex pathways involved in multiple genes to generate high-value oil products in this easily transformable camelina host. Synthetic scaffolds could provide a modular and highly flexible tool for rationally organizing multiple function-related enzymes in a controllable manner (Pröschel et al., 2015). It is envisioned that *C. sativa* can be developed as the adaptable chassis plant for commercial production of high-valued compounds by synthetic biology tools.

As CRISPR/Cas9 advance for targeted genome editing in recent years (Wolter et al., 2019), this tool makes it possible to introduce the assembled transgene modules into specific loci in the host crop, leading to an enhancement in the predictability of transgenesis and metabolic outcomes. Like the cases discussed above, CRISPR/Cas9 was used to “knock out” or disable the target gene in the competing pathways so as to direct metabolic flux toward the desired route. Our laboratory used this technology to construct a library of gene-related mutants associated with the oil metabolism in camelina seeds and obtained various mutants with a single copy of the gene. This shows that the CRISP/Case9 technology has multi-faceted application values in *C. sativa* to overcome the tripled orthologous genes. Undoubtedly, advances in synthetic biology could make it more effectively to perform modular transgene assembly and its targeting insertion into the host genome. Improvements in genome editing could allow the accurate optimization of the host metabolism for the desired pathway. Combination of these two technologies could significantly increase the efficiency of plant lipid redesign and pathway assembly.

In order to obtain various high-valued lipid products, it is also required to redesign the enzyme with improved activities or specific properties. Several approaches can be used to modify or generate novel enzymes necessary for the target lipid synthesis and accumulation, including directed protein mutation, codon-optimization and post-translation modifications. Finally, re-design of metabolic pathways for enriching novel and high-valued lipids should be combined with other breeding programs in camelina such as increasing yield and seed oil content, and improving tolerance to various stress conditions. By doing this, new types of engineered camelina stacking the target FAs and other improved agronomic traits will be created, greatly promoting sustainable production of the designed oil and its commercialization.

## Conclusions

Camelina, an important oil crop in the world, is being developed as an ideal “platform crop” for production of high-valued lipids/oils by metabolic engineering. This review described genetic improvements of the seed oil quality in camelina, particularly focusing on the metabolic pathway assembly of novel fatty acids/oils with multiple nutritional and industrial applications. Several successful examples were provided here to show how lipid biosynthesis in camelina seeds can be redesigned to enable the high accumulation of the target oils that have beneficial functional groups or properties. These target products include acTAGs, hydroxylated FAs, medium chain FAs, ω-3-LC-PUFAs and ω-7 FAs. Technological strategies used in this regard include introduction of a new pathway by synergistical overexpression of multiple genes, blocking the competitive pathway by RNAi-mediated suppression or genomic editing, and optimizing the related metabolic pathways using mutant enzymes. New knowledge and technology, particularly synthetic biology and targeting genome editing will be collectively employed in this field to achieve a more complete orchestration of fatty acid synthesis and metabolism as well as sustainable yields of target lipids in camelina and other oil crop seeds although some significant challenges remain to address.

## Author Contributions

LY was involved in the review writing. RL was involved in manuscript refinement. All authors read and approved the final manuscript.

## Funding

This work was financially supported by grants from the National Natural Science Foundation of China (Grant No. 31801400), Sci-Tech Key Research Project of Jinzhong City (Grant No. Y182009), “1331 project” for key innovation team of Jinzhong University (Grant No. jzxycxtd2019009), University Sci-Tech Innovation Project of Shanxi Province (Grant No. 2016171), Basic Research for Application Project of Shanxi Province (Grant No. 201601D202060), Coal-based Key Sci-Tech Project of Shanxi Province (Grant No. FT-2014-01), and the Key Project of The Key Research and Development Program of Shanxi Province, China (Grant No. 201603D312005).

## Conflict of Interest

The authors declare that the research was conducted in the absence of any commercial or financial relationships that could be construed as a potential conflict of interest.
